# EPR study of NO radicals encased in modified open C_60_ fullerenes

**DOI:** 10.5194/mr-1-197-2020

**Published:** 2020-09-10

**Authors:** Klaus-Peter Dinse, Tatsuhisa Kato, Shota Hasegawa, Yoshifumi Hashikawa, Yasujiro Murata, Robert Bittl

**Affiliations:** 1 Freie Universität Berlin, Fachbereich Physik, Arnimallee 14, 14195 Berlin, Germany; 2 Institute for Chemical Research, Kyoto University, Uji, Kyoto 611-0011, Japan

## Abstract

Using pulsed electron paramagnetic resonance (EPR) techniques, the low-temperature magnetic properties of the NO radical being confined in two different modified open $C_{60}$ -derived cages are determined. It is found that the smallest principal g value $g_{3}$ , being assigned to the axis of the radical, deviates strongly from the free electron value. This behaviour results from partial compensation of the spin and orbital contributions to the $g_{3}$ value. The measured $g_{3}$ values in the range of 0.7 yield information about the deviation of the locking potential for the encaged NO from axial symmetry. The estimated 17 meV asymmetry is quite small compared to the situation found for the same radical in polycrystalline or amorphous matrices ranging from 300 to 500 meV. The analysis of the temperature dependence of spin relaxation times resulted in an activation temperature of about 3 K, assigned to temperature-activated motion of the NO within the modified open $C_{60}$ -derived cages with coupled rotational and translational degrees of freedom in a complicated three-dimensional locking potential.

## 1 Introduction

In a series of recent publications, the Kyoto Group has shown that it is possible to encapsulate small and even reactive molecules in a modified $C_{60}$ cage with tailored entrance and exit holes [Bibr bib1.bibx6]. Using such designer-type open cages instead of closed structures creates a new route for the preparation of interesting compounds. The family of endohedral fullerenes with closed carbon cages like N@ $C_{60}$ [Bibr bib1.bibx16], He@ $C_{60}$ [Bibr bib1.bibx21], $H_{2}$ @ $C_{60}$ [Bibr bib1.bibx10], $H_{2}O$ @ $C_{60}$ [Bibr bib1.bibx12], HF@ $C_{60}$ [Bibr bib1.bibx11], and ${\mathrm{CH}}_{4}$ @ $C_{60}$ [Bibr bib1.bibx1], as well as $C_{82}$ [Bibr bib1.bibx23] based metallo-endohedrals, can thus be expanded significantly. It has been shown that these new compounds can be stable under ambient conditions, allowing easy handling. If encapsulated molecules are paramagnetic, as in the case of ${}^{3}O_{2}$ or ${}^{2}\mathrm{NO}$ , electron paramagnetic resonance (EPR) is the method of choice for elucidating their properties. This not only allows determination of the stationary-spin Hamilton parameters, but furthermore allows detection of dynamic properties arising from internal dynamics or motion of the compound as a whole. In the case of La@ $C_{82}$ for instance, it was possible to conclude from an analysis of two-dimensional EXCSY spectra that the metal ion is rigidly locked to the inside surface of the carbon cage [Bibr bib1.bibx19]. In the present case of an encapsulated NO radical it was concluded from the broad variance of its principal g matrix values [Bibr bib1.bibx6] that even at low temperatures the radical is not fixed to a particular site. It was remarkable that the very small value quoted for the axial component [Bibr bib1.bibx6] of 0.225 deviates significantly from the value determined for NO radicals trapped in a single crystal host [Bibr bib1.bibx20] or NO radicals adsorbed in zeolites [Bibr bib1.bibx18]. This very small value of $g_{3}=0.225$ , deduced by an analysis of a continuous wave (cw) measurement, necessitated confirmation by pulse EPR experiments, better suited for the study of very broad spectra. Although a $T_{2}$ variation as a function of the external field can distort the shape of a pulse-derived spectrum to some extent, difficulties in detecting extremely broad spectra with virtually absent changes within the typically achievable $B_{0}$ modulation amplitudes in cw EPR can lead to misinterpretations, in particular if the supposed spectrum extends a factor of 2 beyond the possible acquisition range. So far, neither relaxation nor nitrogen hyperfine data were reported, which might be important for a full characterization of the compound. It was the aim of the present study to obtain by multi-frequency EPR and ENDOR techniques a complete spin Hamiltonian parameter set for the encapsulated radical. In addition, the anticipated effects of a non-spherical cage potential on the radical are explored, and effects due to the structural modification of the cage are studied.

## 2 Experimental part

### 2.1 Sample preparation

NO radicals trapped in two slightly different modified $C_{60}$ cages, $C_{82}H_{28}N_{3}O_{5}S$ and $C_{82}H_{32}N_{3}O_{5}S$ , were studied, in the following abbreviated by NO@C60-OH1 and NO@C60-OH3, respectively (see Fig. [Fig Ch1.F1] for NO@C60-OH1 and Fig. [Fig App1.Ch1.S1.F8] for NO@C60-OH3). The notation indicates the two different orifices with one and three OH groups, respectively. NO@C60-OH1 was prepared as described in [Bibr bib1.bibx6] and NO@C60-OH3 combining the procedures described in [Bibr bib1.bibx8] and [Bibr bib1.bibx6]. NO@C60-OH1 and NO@C60-OH3 were dissolved in ${\mathrm{CS}}_{2}$ in 2.5 and 10 mM concentrations and sealed in quartz tubes for EPR spectroscopy.

**Figure 1 Ch1.F1:**
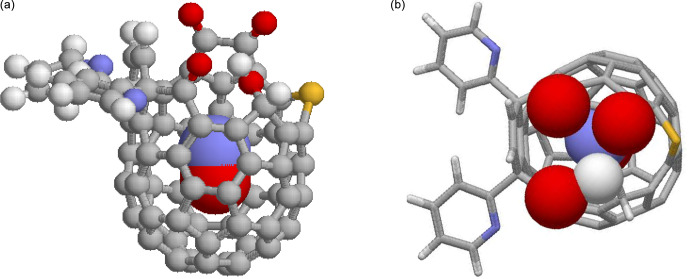
DFT-optimized structure of NO@C60-OH1 with N “up”. **(a)** Ball-and-stick representation of the modified $C_{60}$ cage and van der Waals spheres of the caged NO with carbon (grey), hydrogen (white), nitrogen (blue), oxygen (red), and sulfur (yellow). **(b)** Top view on the orifice with stick representation of the cage except van der Waals spheres for the oxygen and hydrogen atoms of the orifice and the caged NO.

### 2.2 EPR spectroscopy

For pulsed EPR and ENDOR measurements at S- and X-band mw frequencies (3.4 and 9.8 GHz), various setups were employed. Echo-detected 9.8 GHz EPR measurements at low temperatures were conducted on Bruker ElexSys E580 and E680 instruments equipped with Oxford CF935 helium cryostats using Bruker MD4 Flexline ENDOR probe heads. Field-swept echo-detected EPR spectra (FSE) at 9.8 GHz were recorded using a two-pulse “Hahn-echo” sequence (20–300–40 ns) at temperatures of 3.6 to 12 K, yielding absorption-type spectra. Transient nutation measurements at 9.8 GHz were conducted by applying a PEANUT [Bibr bib1.bibx25] pulse sequence with a π/2 pulse length of 8 ns, a delay time τ of 130 ns and a high turning angle (HTAx) pulse of 4096 ns. Phase inversion time within the high turning angle (HTAx) pulse was incremented by 2 ns starting with an initial inversion after 16 ns. ENDOR spectra were recorded by applying either a Mims pulse sequence with π/2 pulses of 20 ns, a delay time τ of 200 ns and a rf π pulse length of 15 µ s, or a Davies pulse sequence with pulse settings 40–30 000–20–200 ns and a RF pulse length of 25 µ s. FSE data at a microwave frequency of 3.4 GHz (S-band) were obtained again using a Bruker ElexSys E680 system with an additional S-band accessory including a Bruker Flexline probe head with a split-ring resonator employing a pulse timing of 32–500–64 ns. FSE and ENDOR spectra were fitted by the EasySpin [Bibr bib1.bibx24] “esfit” routine using the “pepper” and “salt” simulation routines.

### 2.3 Quantum chemical calculations

Optimization of the structure of the compounds NO@C60-OH1 and NO@C60-OH3, with replacement of the 6-t-Butylpyridin-2-yl groups with 2-pyridyl groups, has been performed using Gaussian [Bibr bib1.bibx4] at the HPC center of FU Berlin. DFT calculations were performed using the 6-311++ basis set with UB3LYP exchange. Structures derived for nitrogen in the “up” orientation (with respect to the orifice) are depicted in Figs. [Fig Ch1.F1] and [Fig App1.Ch1.S1.F8]. The difference in total energies for “up” and “down” orientations of the trapped radical was 22.6 meV for NO@C60-OH1, somewhat larger than the value (8 meV) published earlier [Bibr bib1.bibx6], which might be caused by use of a different basis set. For NO@C60-OH3 we calculated 40.2 meV.

## 3 Results and discussion

### 3.1 Multi-frequency EPR data

EPR data published previously by [Bibr bib1.bibx6] for NO@C60-OH1 were obtained in cw mode at a microwave frequency of 9.56 GHz. Spectra measured at 3.45 and 9.76 GHz using the FSE technique are depicted in Fig. [Fig Ch1.F2]. The published g matrix parameter set (see Table [Table Ch1.T1]) obtained by spectral simulation of the cw spectrum is characterized by an extreme g anisotropy. The values determined by fitting the FSE spectra confirm the two larger g matrix parameters; however, they deviate significantly with respect to the pseudo-axial $g_{3}$ parameter.

**Figure 2 Ch1.F2:**
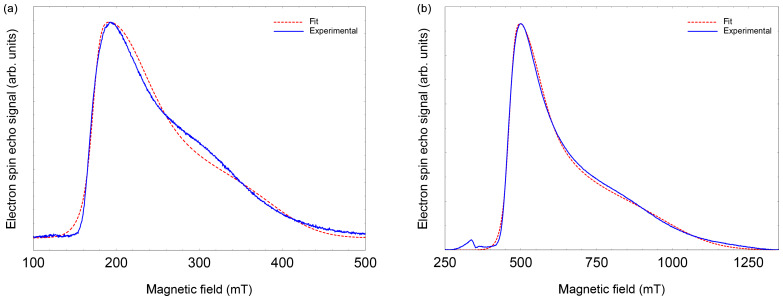
FSE spectra of NO@C60-OH1 ( T=5 K) with best fits. **(a)** S-band (3.5 GHz, 10 mM/ ${\mathrm{CS}}_{2}$ ). **(b)** X-band (9.7 GHz, 2.5 mM/ ${\mathrm{CS}}_{2}$ ). For fitting a set of nitrogen hyperfine tensor parameters was used, determined by ENDOR (see below).

**Table 1 Ch1.T1:** Fit-determined g matrix data of both compounds (gStrain fit parameters are listed in brackets). Previously published values [Bibr bib1.bibx6] are given for comparison. Level splittings Δ deduced from the deviation of the pseudo-axial $g_{3}$ parameter from $g_{e}$ are also shown.

Sample	ν (GHz)	cw/FSE	$g_{1}$	$g_{2}$	$g_{3}$	Δ (meV)
NO@C60-OH1	3.45	FSE	1.438(0.007)	1.225(0.399)	0.646(0.134)	15.9
NO@C60-OH1	9.76	FSE	1.482(0.002)	1.350(0.275)	0.679(0.182)	16.9
NO@C60-OH3	3.45	FSE	1.480(0.012)	1.212(0.602)	0.725(0.129)	17.8
NO@C60-OH3	9.76	FSE	1.527(0.002)	1.422(0.287)	0.767(0.173)	19.7
NO@C60-OH1	9.57	cw	1.488	1.320	0.225	

We quote no error margins, because a large g strain value is obtained for the $g_{3}$ value using the “esfit” routine (EasySpin; [Bibr bib1.bibx24]). The pseudo-axial principal parameters $g_{3}=0.646$ and 0.679, respectively, are still found to be very small compared to $g_{3}=1.7175$ for the same compound trapped in a crystal [Bibr bib1.bibx20] or $g_{3}=1.888$ when incorporated into a zeolite [Bibr bib1.bibx18], but rendering the g matrix substantially less anisotropic compared to the data in [Bibr bib1.bibx6]. For further confirmation of the g matrix parameter set determined by fitting the FSE spectra, we also performed a PEANUT experiment [Bibr bib1.bibx25], probing the Rabi nutation frequency as a function of $B_{0}$ (see below).

Parameters determined for the NO@C60-OH3 compound are also listed in Table [Table Ch1.T1]. Spectra are shown in Fig. [Fig App1.Ch1.S1.F9] in Appendix [App App1.Ch1.S1]. Also for this compound with a slightly modified cage a similar set is observed, the fit parameters changing slightly towards larger values compared to those found for the OH1 compound. Even the slight difference in cage structure apparently is influencing the g matrix values. However, no prominent features of anticipated magnetic interaction between encapsulated NO radicals within the intermolecular hydrogen-bonded dimeric triply hydroxylated $C_{60}$ -derived cages were observed.

Because of the rather large deviation of the $g_{i}$ parameters from the free electron value and the large anisotropy of g , a significant variation of the nutation frequency was expected as a function of orientation. If by orientation selection a particular g principal position is chosen, the two remaining g parameters determine the nutation frequency. As shown in Fig. [Fig Ch1.F3], all Rabi frequencies are smaller than the reference value determined by a standard coal sample and increase towards the high field spectral range. In Fig. [Fig Ch1.F3], the expected nutation frequency distributions [Bibr bib1.bibx25] are indicated by dashed vertical lines at the g principle values using the values for NO@C60-OH3 at X-band in Table [Table Ch1.T1]. The agreement is quite convincing, and a very small $g_{3}$ parameter as deduced earlier can be excluded, since it would lead to much smaller nutation frequencies down to ≈3.7 MHz in the perpendicular orientations ( $g_{1}$ and $g_{2}$ region, 500 mT region) of the radical. Thus, the small value of $g_{3}=0.225$ [Bibr bib1.bibx6] is probably caused by overestimating the flat high field part of the cw spectrum in the simulation.

**Figure 3 Ch1.F3:**
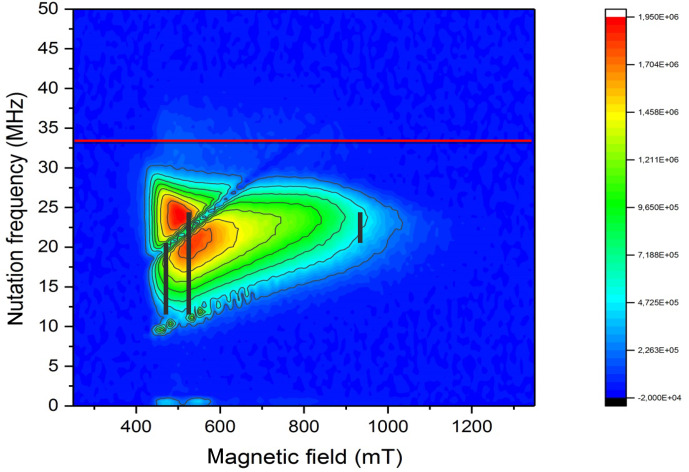
PEANUT spectrum of NO@C60-OH3 measured at 5 K. The red line indicates the reference frequency measured for a coal sample with isotropic g=2 . The black vertical lines indicate the expected nutation frequency distributions [Bibr bib1.bibx25] at the three principal g values for NO@C60-OH3 in Table [Table Ch1.T1] (X-band).

It should be noted that the g matrix parameters of the encapsulated NO radical deviate much more from the free electron value $g_{e}=2.0023$ compared to data reported for situations when the radical is either trapped in a crystal [ g=(1.9740(7),1.9766(7),1.7175(4)) ] [Bibr bib1.bibx20], adsorbed at the surface of metal oxides [ g=(1.97,1.97,1.91) ] [Bibr bib1.bibx13], or incorporated into a zeolite [ g=(2.001,1.996,1.888) ] [Bibr bib1.bibx18]. This clearly indicates that the orbital momentum of the radical is much less quenched in the $C_{60}$ -derived cages. Following the idea that partial quenching of the orbital angular momentum is caused by lifting of the degeneracy between the antibonding ${}^{2}\pi_{x}$ and ${}^{2}\pi_{y}$ orbitals, the energy splitting Δ between these orbitals can be estimated by the pseudo-axial value of the NO g matrix [Bibr bib1.bibx20]:

1 $$g_{3}=g_{e}-2\lambda L/(\lambda^{2}+\Delta^{2}{)}^{1/2}.$$

Here, λ is the spin-orbit coupling constant (123.16 cm ${}^{-1}$ for NO; [Bibr bib1.bibx9]), Δ defines the crystal-field splitting of the ${}^{2}\pi_{x}$ and ${}^{2}\pi_{y}$ orbitals, and L is a correction to the angular momentum along z caused by the crystal field. L is equal to 1 for a free molecule. A change in L represents a modification of the molecular wave function by the crystal field. It should be noted, however, that in previous studies [Bibr bib1.bibx27] no significant deviations from 1 were observed. The highly nonlinear dependence of $g_{3}$ on Δ is depicted in Fig. [Fig App1.Ch1.S1.F10] (Appendix [App App1.Ch1.S1]). Using Eq. ([Disp-formula Ch1.E1]), a level splitting of approximately 17 meV (200 K) is determined from $g_{3}\approx 0.7$ for NO@C60-OH1 and 20 meV from $g_{3}\approx 0.8$ for NO@C60-OH3 (see Table [Table Ch1.T1]).

The lifting of the ${}^{2}\pi_{x}{/}^{2}\pi_{y}$ degeneracy is not unexpected considering the observation of a finite zero-field splitting (ZFS) for ${}^{3}O_{2}$ in a cage with $C_{1}$ symmetry [Bibr bib1.bibx5]. In this study the potential barrier for librational motions of ${}^{3}O_{2}$ was estimated as 398 cm ${}^{-1}$ (49 meV) by measuring the shift of its principal ZFS component with respect to the value of the free molecule. The size of this potential barrier is on the same order of magnitude as the one calculated here for NO. The lifting of degeneracy leads to a deviation of the orbitals from two fully circular symmetric angular momentum eigenstates with opposite momentum to two orthogonal elliptic orbitals not being angular momentum eigenstates, but with non-vanishing angular momentum expectation values. With a 200 K level splitting only one of the orbitals is occupied at 5 K and rotation of the molecule corresponds to transitions from one to the other eigenstate, which should be impossible due to the large level splitting. Nevertheless, the remaining angular momentum expectation value gives rise to the very small $g_{3}$ value. The splitting is much less than values found for ${}^{2}\mathrm{NO}$ and ${}^{2}O_{2}^{-}$ trapped in crystals, on surfaces, or in zeolites, which range from 300 to 500 meV.

**Figure 4 Ch1.F4:**
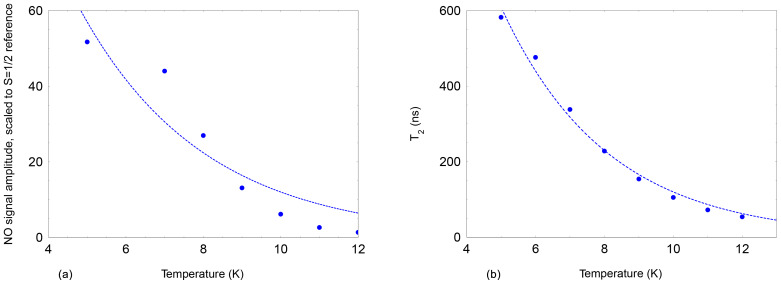
Echo-detected signal of NO@C60-OH3 (480 mT, 9.7 GHz, 2.5 mM/ ${\mathrm{CS}}_{2}$ , 200 ns pulse separation) as a function of temperature. The signal intensity is scaled to the intensity of a field-separated $g\approx g_{e}$ signal from an unidentified S=1/2 species following the Curie law. An exponential temperature dependence is assumed for the fit (dashed line) with a decay constant of 3 K. **(b)** Temperature dependence of the spin echo decay constant $T_{2}$ of NO@C60-OH3 (480 mT, 9.7 GHz, 2.5 mM/ ${\mathrm{CS}}_{2}$ ). The faster decay constant with larger weight is shown in cases where the time traces required a bi-exponential fit. Again an exponential temperature dependence is assumed for the dashed line, with a decay constant of 3.1 K.

The ${}^{2}\pi_{x}$ and ${}^{2}\pi_{y}$ level splitting is of the same order of magnitude as the energy difference for the “up” and “down” orientation of the NO radical with respect to the cage opening calculated earlier [Bibr bib1.bibx6] and also found in this study. For “up”/“down” axis reorientation a factor 10 larger barrier was found. Considering the additional degree of freedom of hindered rotation about the axis of the radical with an unknown transition barrier, this gives rise to a complicated three-dimensional orthorhombic potential energy surface. It is not surprising that under these conditions the EPR signal can be detected only at very low temperatures. The temperature dependence of the NO FSE signal (X-band) was measured relative to an unidentified stable $S=1/2,g\approx g_{e}$ species in the sample and is shown in Fig. [Fig Ch1.F4] (left). The NO signal decreases much faster upon temperature increase than according to the Curie law since a dramatic signal loss relative to the reference signal is observed. This strong additional signal decay of the NO radical beyond the Curie law can be described by an activation temperature of about 3 K.

The dramatic loss of signal intensity by a factor 50 in the narrow temperature range of 5 to 12 K is indicative of a decrease in $T_{2}$ . This was confirmed by measuring the two-pulse echo decay constant $T_{2}^{*}$ at the peak signal position. Its temperature dependence could be fitted assuming exponential temperature dependence with an activation temperature of 3.1 K as shown in Fig. [Fig Ch1.F4] (right).

**Figure 5 Ch1.F5:**
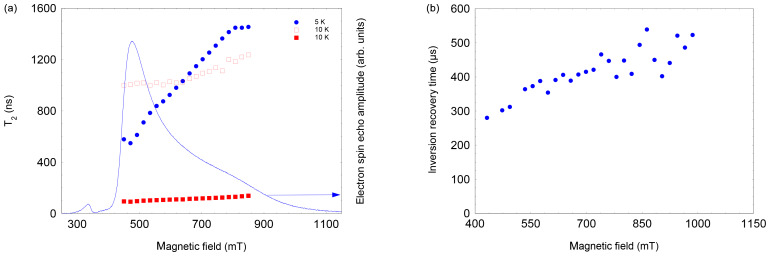
**(a)** Field and temperature dependence of the two-pulse echo decay of NO@C60-OH3. The 5 K data set could be satisfactorily fitted assuming a single exponential; the 10 K data required a bi-exponential fit. Both components were of similar magnitude, with the shorter $T_{2}$ larger in amplitude. **(b)** Field dependence of $T_{1}$ of NO@C60-OH3, measured using an inversion recovery pulse sequence at 3.6 K.

Measuring the field dependence of $T_{2}^{*}$ at different temperatures supports the simple model of a restricted rotation. As shown in Fig. [Fig Ch1.F5] left, at 5 K the $T_{2}^{*}$ values increase from 600 to 1500 ns, with probing radicals changing from perpendicular to parallel orientation. This can be taken as evidence that small angle librations around the long axis are activated at this temperature, whereas long-axis reorientations are still prevented at this temperature. In contrast, at 10 K this restriction is no longer valid, shortening the echo decay accordingly for the full field range; i.e. librations about all molecular axes occur.

This hypothesis is also supported by the observation that $T_{1}$ , determined by inversion recovery, also increases significantly at T=3.6 K when moving from perpendicular to parallel orientation (see Fig. [Fig Ch1.F5], right). This field dependence of $T_{1}$ leads even at 3.6 K to a noticeable change in the FSE pattern if the pulse repetition time is not sufficiently long (see Fig. [Fig App1.Ch1.S1.F11], Appendix [App App1.Ch1.S1]). While $T_{1}$ and $T_{2}$ show a significant temperature dependence, the spectral shape, and thus the g parameters, are virtually unaffected within the temperature range of 3.6 to 12.5 K (see Table [Table App1.Ch1.S2.T4], Appendix [App App1.Ch1.S2]).

**Figure 6 Ch1.F6:**
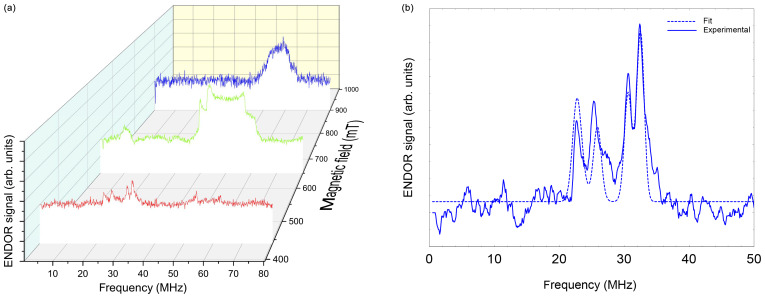
**(a)** Davies ENDOR spectra of NO@C60-OH1 ( T=5 K, 10 mM/ ${\mathrm{CS}}_{2}$ ) measured as a function of $B_{0}$ . Spectra are corrected with respect to different accumulation times for better comparison of spectral patterns. The Davies ENDOR pulse sequence (40–30 000–20–200–40 ns, 25 µ s rf pulse) was identical for all spectra. **(b)** ENDOR spectrum of NO@C60-OH1 measured at 440 mT (see panel **a**) together with simulation.

Loss of the cw EPR signal intensity at temperatures above 80 K was also reported in [Bibr bib1.bibx7]. Since the cw signal intensity is not affected by $T_{2}^{*}$ , the NO signal could be detected in cw mode up to 40 K [Bibr bib1.bibx7], with a much smaller decrease from 5 to 20 K than observed in our pulsed EPR study probing the echo signal with a two-pulse sequence. The low activation temperature of 3 K ( ∼0.3 meV) has to be compared to the much larger values found in the case of N@ $C_{60}$ and P@ $C_{60}$ , in which a well-defined potential of spherical or axial symmetry leads to degenerate vibrational levels of the translational degree of freedom of encapsulated atoms in the range of 8 to 16 meV [Bibr bib1.bibx17], respectively. The partially opened cage resembles more the situation in the $C_{70}$ cage by providing a nearly axial potential. Assuming that vibration along this preferred axis is lowest in energy and taking into account the larger mass of the radical, a vibrational eigenfrequency of about 5 meV for the center of mass (CM) of the radical would be expected, which is still more than 1 order of magnitude larger than the experimental value. In contrast to encapsulated atoms, we also have to consider for the NO case a librational mode of the radical with respect to the cage axis. In a study of $H_{2}$ encapsulated in $C_{60}$ or $C_{70}$ , the eigenstates of $H_{2}$ were determined numerically by invoking the appropriate five-dimensional potential surface, describing translational and rotational degrees of freedom [Bibr bib1.bibx26]. Lacking numerical values for the potential surface in our more complicated case, it is only possible to estimate typical values for the librational mode by approximating the interconversion between “up”/“down” (its z axis) of the radical axis in a potential well of 80 meV (645 cm ${}^{-1}$ ) [Bibr bib1.bibx6] as a torsional oscillator. Converting the 80 meV rotational barrier into a torsion spring constant for librations of κ=40 meV and using $\omega =\sqrt{\kappa /\theta }$ with the moment of inertia θ of NO, we arrive at a characteristic mode energy for the libration of about 4 meV (about 40 K), which is substantially larger than the experimentally observed activation temperature. However, when including transverse degrees of freedom for axis reorientation, it is not unlikely that the characteristic mode energies might further be reduced towards the experimental value.

**Table 2 Ch1.T2:** Hyperfine parameters determined by fitting Davies ENDOR spectra measured under orientation selection conditions providing the best resolution. For an assignment of signs, see text. n.d.: not determined.

Sample	$A_{1}$	$A_{2}$	$A_{3}$	$Q_{1}$	$Q_{2}$	$Q_{3}$
	(MHz)	(MHz)	(MHz)	(MHz)	(MHz)	(MHz)
NO@C60-OH1	-55.3	n.d.	+122 .6	2.47	n.d.	n.d.
NO@C60-OH3	n.d.	n.d.	+124.1	n.d.	n.d.	1.1

**Table 3 Ch1.T3:** Hyperfine parameters calculated for NO@C60-OH1 and NO@C60-OH3 in their “up” configuration using Gaussian G16/A03 (G16/A03, B3LYP, 6-311++). The calculated values for the “down” orientation differ by less than 3 %.

Sample	$A_{1}$	$A_{2}$	$A_{3}$	$Q_{1}$	$Q_{2}$	$Q_{3}$
	(MHz)	(MHz)	(MHz)	(MHz)	(MHz)	(MHz)
NO@C60-OH1	-25.5	-23.5	+90.2	-1.48	+0.22	+1.26
NO@C60-OH3	-25.4	-23.1	+90.5	-1.48	+0.22	+1.26

### 3.2 ENDOR data

Orientation-selective ENDOR spectra of NO@C60-OH1 were measured at 9.7 GHz. As depicted in Fig. [Fig Ch1.F6] (left), the center of lines shifts towards higher frequency when changing the observation field position from the lowest to highest edges of the absorption pattern. The frequency position at the low field side of the spectrum and the magnitude of the shift is inconsistent with proton hfi but is indicative of a dominant dipolar ${}^{14}N$ hfi, allowing simple determination of $A_{i}$ for the extreme field positions. For a determination of dipolar and quadrupolar hfi parameters, observation field values at the low and high ends of the FSE spectrum were chosen, anticipating that g matrix and hfi tensor axes are collinear. Best ENDOR resolution is obtained at the low field edge, allowing determination of some hfi parameters by fitting, as shown in Fig. [Fig Ch1.F6] (right).

At this field position a consistent fit is obtained by only fixing the nuclear Larmor frequency to its field-determined value. At the high field edge no line quartet is observed for this compound. The broad pattern, however, is consistent with the result of a spectral simulation, shown in Fig. [Fig Ch1.F7], using a parameter set completed with the nqi parameter of NO@C60-OH3, being better resolved at the high-field edge of the ENDOR pattern. It should be noted that no simple pattern is expected for the intermediate field range because of significant g strain. For this reason fit values are only quoted assigned to the $g_{1}$ and $g_{3}$ axis directions. No information about the signs of hfi parameters can be deduced from the experimental spectra. The assignments given in Table [Table Ch1.T2] are tentatively made by invoking the calculated hfi constants (see Table [Table Ch1.T3]). Although not in very good quantitative agreement with the experiment, the calculated small isotropic hfi ( +15 MHz) necessitates assignment of a negative sign to $A_{1}$ . Lacking spectral resolution when probing at the high field edge due to the large $g_{3}$ strain, the center of gravity still gives a reliable value for the large dipolar hfi for both compounds. The absent spectral resolution, even when observing at the van Hove singularities of the FSE spectrum, could result from a simultaneous presence of “up”/“down” configurations as observed in X-ray crystallography, with slightly different hfi parameters.

**Figure 7 Ch1.F7:**
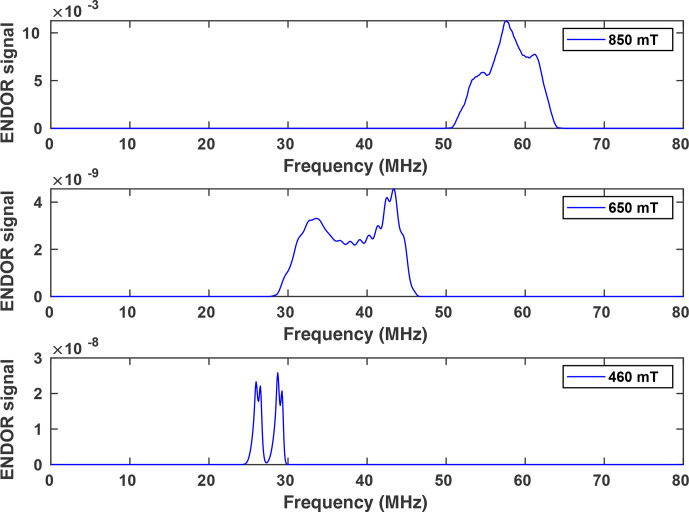
Simulated ENDOR spectra of NO@C60-OH1, using parameters listed in Table [Table Ch1.T2].

## 4 Conclusions

Using various EPR techniques, the spin Hamiltonian parameters for the encapsulated NO radical are determined. The radical, being confined in $C_{60}$ -derived cages, exemplifies the transition between a free molecule in isotropic potential and being fixed by a rigid confinement. The NO radical is particularly suited for such an investigation, since the g factor of the free molecule in its ${}^{2}\Pi_{1/2}$ rotational ground state will change between zero [Bibr bib1.bibx15] to a g matrix, in which all parameters are close to the free electron value for the rigidly localized radical [Bibr bib1.bibx2]. In case the axial molecular symmetry is maintained by the environment allowing free rotation about its axis, the g parameter $g_{3}$ , being assigned to the NO bond axis, is predicted to vanish. The measured value $g_{3}=0.77(5)$ is indicative of an intermediate situation of the radical and yields information about the locking potential's deviation from axial symmetry. This 17 meV asymmetry as found here is quite small compared to the situation in polycrystalline or amorphous matrices ranging from 300 to 500 meV. The analysis of the spin relaxation times resulted in an activation temperature of about 3 K, assigned to temperature-activated motion of the radical with coupled rotational and translational degrees of freedom in the complicated three-dimensional potential provided by the cage.

Performing ENDOR, the ${}^{14}N$ hyperfine coupling parameters were determined. The experimental values are in fair agreement with predictions from a DFT calculation. The spectral resolution was not sufficient to discriminate between parameter sets expected for the X-ray crystallography-confirmed “up”/“down” configurations of the radical with respect to the orifice of the cage.

The g matrix parameters did not show any temperature dependence in the range of 3.6 to 12 K, in which a dramatic orientation-dependent decrease in $T_{2}^{*}$ is observed. This indicates that the radical is localized, not allowing for excitation of rotational modes about its axis, which would modify the $g_{3}$ value. Apparently only low-energy modes with small amplitude around their equilibrium orientation are excited at these temperatures. It should be noted, however, that the accuracy of the data analysis is high enough to detect a small difference in g parameters using cages with slightly modified orifices. It will be interesting to see in the future whether advanced computational methods will be able to reproduce g matrix and hfi tensor data for this radical in such a complicated potential.

## Figures and Tables

**Figure A1 App1.Ch1.S1.F8:**
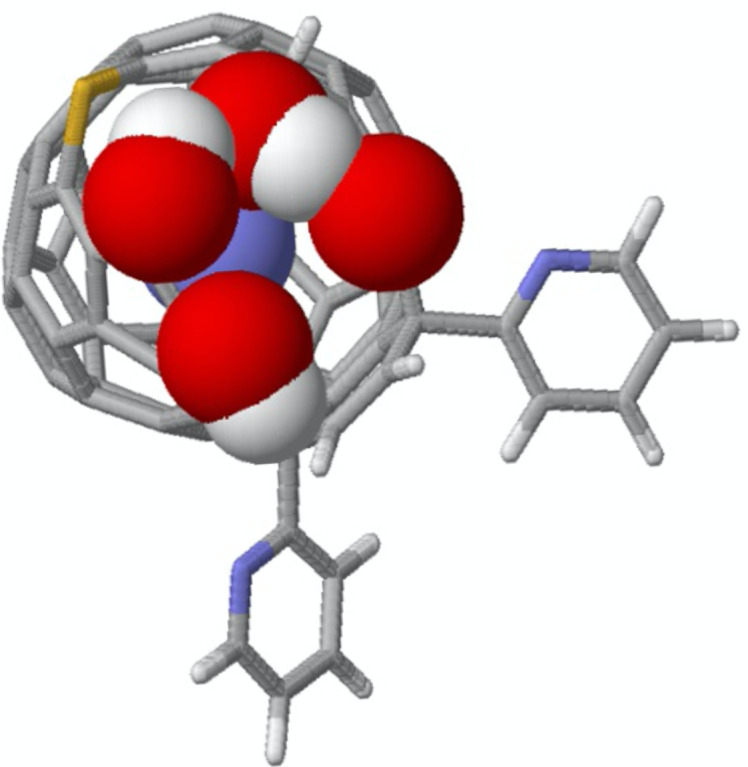
Top view of the DFT-optimized structure of NO@C60-OH3 with N “up”. The modified $C_{60}$ cage is represented by sticks except van der Waals spheres for the oxygen and hydrogen atoms of the orifice and the caged NO – C (grey), H (white), N (blue), O (red), and S (yellow).

**Figure A2 App1.Ch1.S1.F9:**
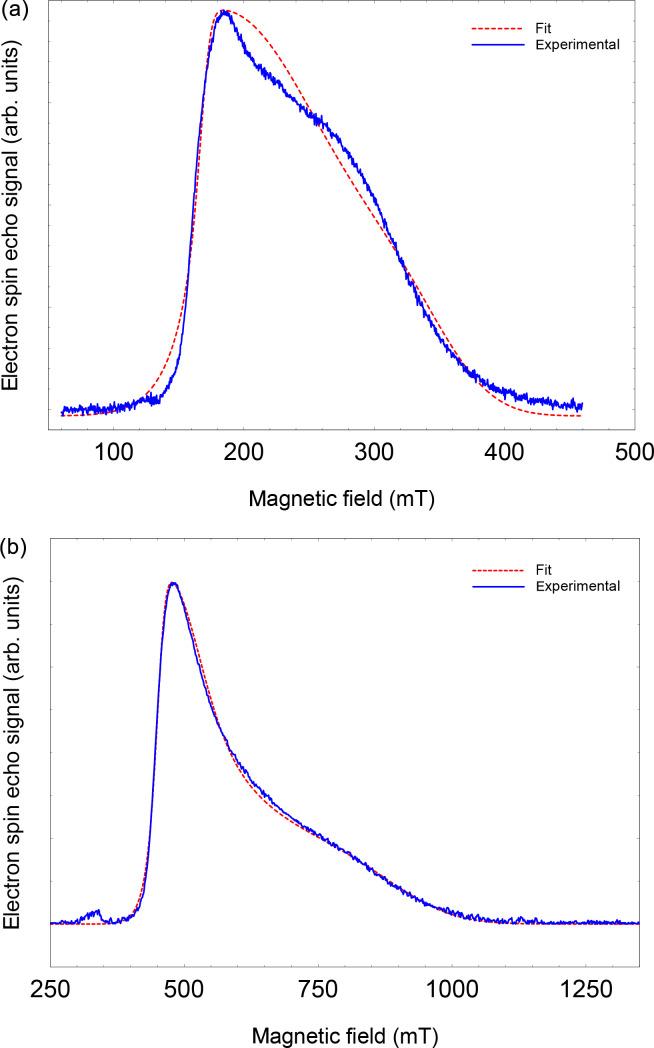
FSE spectra of NO@C60-OH3 ( T=5 K) with best fits. **(a)** S-band (3.5 GHz, 10 mM/ ${\mathrm{CS}}_{2}$ ). **(b)** X-band (9.7 GHz, 2.5 mM/ ${\mathrm{CS}}_{2}$ ).

**Figure A3 App1.Ch1.S1.F10:**
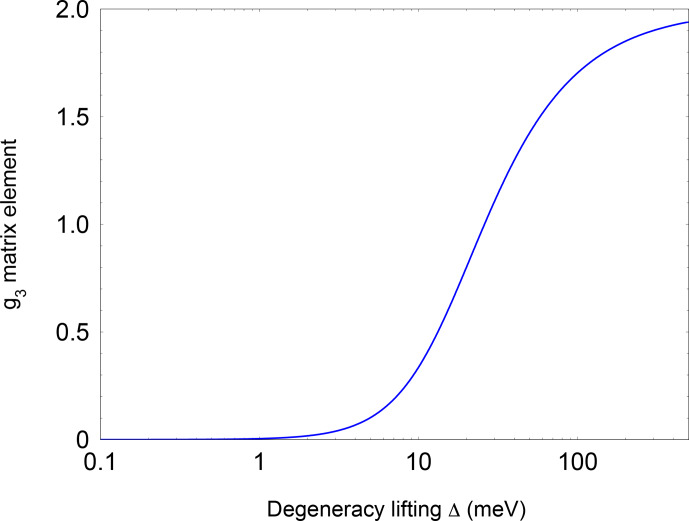
Dependence of the pseudo-axial $g_{3}$ matrix element of the NO radical as a function of ${}^{2}\pi_{x}^{*}$ and ${}^{2}\pi_{y}^{*}$ level splitting.

**Figure A4 App1.Ch1.S1.F11:**
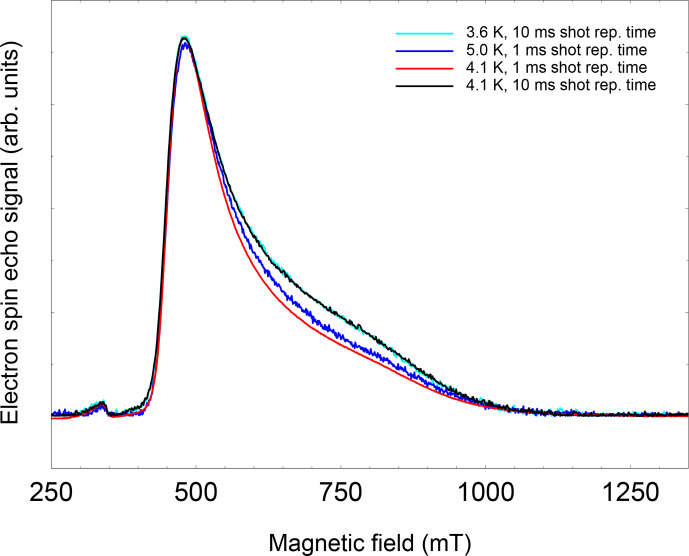
9.7 GHz FSE spectra of NO@C60-OH3 measured at 3.6, 4.1, and 5 K. Using a rather short pulse repetition time (1 ms), the high-field part of the spectrum is partially saturated.

## Data Availability

Experimental data used for the figures and further information are available under DOI https://doi.org/10.17169/refubium-27870 (Dinse et al., 2020).

## Appendix B Additional table

**Table B1 App1.Ch1.S2.T4:** Best fit parameters for the NO@C60-OH3 spectra measured at different temperatures (data not shown).

	$g_{1}$	$g_{2}$	$g_{3}$	$g_{1}$	$g_{2}$	$g_{3}$	Linewidth
				strain	strain	strain	(mT)
5 K, 120 ns	1.527	1.422	0.767	0.002	0.294	0.171	17.4
10 K, 120 ns	1.521	1.420	0.717	0.001	0.348	0.131	12.6
10 K, 300 ns	1.505	1.419	0.699	0.001	0.432	0.139	13.2

## References

[bib1.bibx1] Bloodworth S, Sitinova G, Alom S, Vidal S, Bacanu GR, Elliott SJ, Light ME, Herniman JM, Langley GJ, Levitt MH, Whitby RJ (2019). First Synthesis and Characterization of CH4@C-60. Angew Chem Int Edit.

[bib1.bibx2] Chiesa M, Giamello E, Che M (2010). EPR Characterization and Reactivity of Surface-Localized Inorganic Radicals and Radical Ions. Chem Rev.

[bib1.bibx3] Dinse KP, Kato T, Hasegawa S, Hashikawa Y, Murata Y, Bittl R (2020). Refubium – Freie Universität Berlin Repository.

[bib1.bibx4] Frisch, MJ, Trucks GW, Schlegel HB, Scuseria GE, Robb MA, Cheeseman JR, Scalmani G, Barone V, Petersson GA, Nakatsuji H, Li X, Caricato M, Marenich AV, Bloino J, Janesko BG, Gomperts R, Mennucci B, Hratchian HP, Ortiz JV, Izmaylov AF, Sonnenberg JL, Williams-Young D, Ding F, Lipparini F, Egidi F, Goings J, Peng B, Petrone A, Henderson T, Ranasinghe D, Zakrzewski VG, Gao J, Rega N, Zheng G, Liang W, Hada M, Ehara M, Toyota K, Fukuda R, Hasegawa J, Ishida M, Nakajima T, Honda Y, Kitao O, Nakai H, Vreven T, Throssell K, Montgomery Jr JA, Peralta JE, Ogliaro F, Bearpark MJ, Heyd JJ, Brothers EN, Kudin KN, Staroverov VN, Keith TA, Kobayashi R, Normand J, Raghavachari K, Rendell AP, Burant JC, Iyengar SS, Tomasi J, Cossi M, Millam JM, Klene M, Adamo C, Cammi R, Ochterski JW, Martin RL, Morokuma K, Farkas O, Foresman JB, Fox DJ (2016). Gaussian 16 Revision A.03.

[bib1.bibx5] Futagoishi T, Aharen T, Kato T, Kato A, Ihara T, Tada T, Murata M, Wakamiya A, Kageyama H, Kanemitsu Y, Murata Y (2017). A Stable, Soluble, and Crystalline Supramolecular System with a Triplet Ground State. Angew Chem In Edit.

[bib1.bibx6] Hasegawa S, Hashikawa Y, Kato T, Murata Y (2018). Construction of a Metal-Free Electron Spin System by Encapsulation of an NO Molecule Inside an Open-Cage Fullerene C60 Derivative. Angew Chem Int Edit.

[bib1.bibx7] Hasegawa S, Hashikawa Y, Kato T, Murata Y (2018). supporting information. Angew Chem Int Edit.

[bib1.bibx8] Hashikawa Y, Hasegawa S, Murata Y (2018). A single but Hydrogen-bonded water molecule confined in an anisotropic subnanospace. Chem Commun.

[bib1.bibx9] James TC, Thibault RJ (1964). Spin–Orbit Coupling Constant of Nitric Oxide. Determination from Fundamental and Satellite Band Origins. J Chem Phys.

[bib1.bibx10] Komatsu K, Murata M, Murata Y (2005). Encapsulation of Molecular Hydrogen in Fullerene C60 by Organic Synthesis. Science.

[bib1.bibx11] Krachmalnicoff A, Bounds R, Mamone S, Alom S, Concistre M, Meier B, Kouril K, Light ME, Johnson MR, Rols S, Horsewill AJ, Shugai A, Nagel U, Room T, Carravetta M, Levitt MH, Whitby RJ (2016). The dipolar endofullerene HF@C-60. Nat Chem.

[bib1.bibx12] Kurotobi K, Murata Y (2011). A Single Molecule of Water Encapsulated in Fullerene C60. Science.

[bib1.bibx13] Lunsford JH (1968). Surface interactions of zinc oxide and zinc sulfide with nitric oxide. J Phys Chem.

[bib1.bibx14] Mamone S, Concistre M, Heinmaa I, Carravetta M, Kuprov I, Wall G, Denning M, Lei X, Chen JY, Li Y, Murata Y, Turro NJ, Levitt MH (2013). Nuclear magnetic resonance of hydrogen molecules trapped inside C70 fullerene cages. Chem Phys Chem.

[bib1.bibx15] Mendt M, Pöppl A (2015). The Line Width of the EPR Signal of Gaseous Nitric Oxide as Determined by Pressure and Temperature-Dependent X-band Continuous Wave Measurements. Appl Magn Reson.

[bib1.bibx16] Murphy TA, Pawlik T, Weidinger A, Höhne M, Alcala R, Spaeth JM (1996). Observation of Atomlike Nitrogen in Nitrogen-Implanted Solid C60. Phys Rev Lett.

[bib1.bibx17] Pietzak B, Weidinger A, Dinse KP, Hirsch A (2002). Group V Endohedral Fullerenes: N@C60, N@C70, and P@C60.

[bib1.bibx18] Poeppl A, Rudolf T, Manikandan P, Goldfarb D (2000). W- and X-Band Pulsed Electron Nuclear Double-Resonance Study of a Sodium-Nitric Oxide Adsorption Complex in NaA Zeolites. J Am Chem Soc.

[bib1.bibx19] Rübsam M, Schweitzer P, Dinse KP (1996). Rotational dynamics of metallo-endofullerenes in solution. J Phys Chem.

[bib1.bibx20] Ryzhkov LR, Toscano JP (2005). Crystal Lattice Effects on the Orientation and Orbital Degeneracy of Nitric Oxide Trapped in Nitramine Single Crystals. Cryst Growth Des.

[bib1.bibx21] Saunders M, Jiménez-Vázquez HA, Cross RJ, Mroczkowski S, Gross ML, Giblin DE, Poreda RJ (1994). Incorporation of Helium, Neon, Argon, Krypton, and Xenon into Fullerenes using High Pressure. J Am Chem Soc.

[bib1.bibx22] Shuey RT, Zeller HR (1967). Die elektronische Struktur des 
${}^{2}O_{2}^{-}$
-Zentrums in den Alkalihalogeniden II. Theoretische Betrachtungen. Helv Phys mActa.

[bib1.bibx23] Stevenson S, Rice G, Glass T, Harich K, Cromer F, Jordan MR, Craft J, Hadju E, Bible R, Olmstead MM, Maitra K, Fisher AJ, Balch AL, Dorn HC (1999). Small-bandgap endohedral metallofullerenes in high yield and purity. Nature.

[bib1.bibx24] Stoll S, Schweiger A (2006). A comprehensive software package for spectral simulation and analysis in EPR. J Magn Reson.

[bib1.bibx25] Stoll S, Jeschke G, Willer M, Schweiger A (1998). Nutation-Frequency Correlated EPR Spectroscopy: The PEANUT Experiment. J Magn Reson.

[bib1.bibx26] Xu M, Sebastianelli F, Gibbons BR, Bacic Z, Lawler R, Turro NJ (2009). Coupled translation-rotation eigenstates of 
$H_{2}$
 in C60 and C70 on the spectroscopically optimized interaction potential: Effects of cage anisotropy on the energy level structure and assignments. J Chem Phys.

[bib1.bibx27] Zeller HR, Känzig W (1967). Die elektronische Struktur des 
${}^{2}O_{2}^{-}$
 Zentrums in den Alkalihalogeniden I. Die paramagnetischen und optischen Spektren und ihre Interpretation. Helv Phys Acta.

